# New Onset Coronary Obstruction in Established Nonischemic Heart Failure: A Late Consequence of Radiation

**DOI:** 10.14797/mdcvj.1097

**Published:** 2022-03-14

**Authors:** Salil Kumar, Joe Aoun, Arvind Bhimaraj

**Affiliations:** 1 Houston Methodist DeBakey Heart & Vascular Center, Houston Methodist Hospital, Houston, Texas, US

**Keywords:** radiation heart disease, heart failure, radiation induced coronary artery disease

## Abstract

The column in this issue is provided by Salil Kumar, MD, and Joe Aoun, MD, chief cardiology fellows in the Houston Methodist Cardiology Department, and Arvind Bhimaraj, MD, associate professor of Clinical Cardiology at the Houston Methodist Academic Institute. Dr. Bhimaraj specializes in cardiovascular disease and advanced heart failure and transplantation.

Radiation heart disease is commonly overlooked in patients who have a remote history of radiation therapy. Cardiologists should work to uncover radiation treatment history from patients during clinical encounters. The following case illustrates the importance of uncovering this important history and knowing the time course and different manifestations of radiation-induced cardiovascular disease.

A 49-year-old Caucasian woman presented to our heart failure clinic for a follow-up visit to discuss chest pain. Her history included nonischemic cardiomyopathy presumed secondary to remote chemotherapy, heart failure with reduced ejection fraction (ejection fraction 20-25%), Hodgkin’s lymphoma in remission (20 years ago) after treatment with chemotherapy and chest radiation, and breast cancer (5 years ago) managed with double mastectomy and chemotherapy.

The patient stated that her chest discomfort started 1 year ago. It was substernal and intermittently radiating to her left shoulder, with pain intensity and frequency waxing and waning. The discomfort worsened with exertion and improved with rest. She had a recent acute congestive heart failure exacerbation with significantly worsened chest pain that improved with oral diuretics.

No record was available of the exact chemotherapy and radiation dosages given for her Hodgkin’s lymphoma or breast cancer. A coronary computed tomography angiography (CCTA) done about 8 months prior showed moderate coronary atherosclerosis with moderate stenosis of the left main coronary artery, which was not deemed clinically responsible for her angina.

We proceeded with selective coronary angiography due to her typical symptoms for coronary atherosclerotic vascular disease, which demonstrated a severe (95%) ostial left main disease.

What would you do next?

Due to the low Syntax score, the interventionalist performed left main stenting guided by intravascular ultrasound (***Figure 1***).

**Figure 1:**
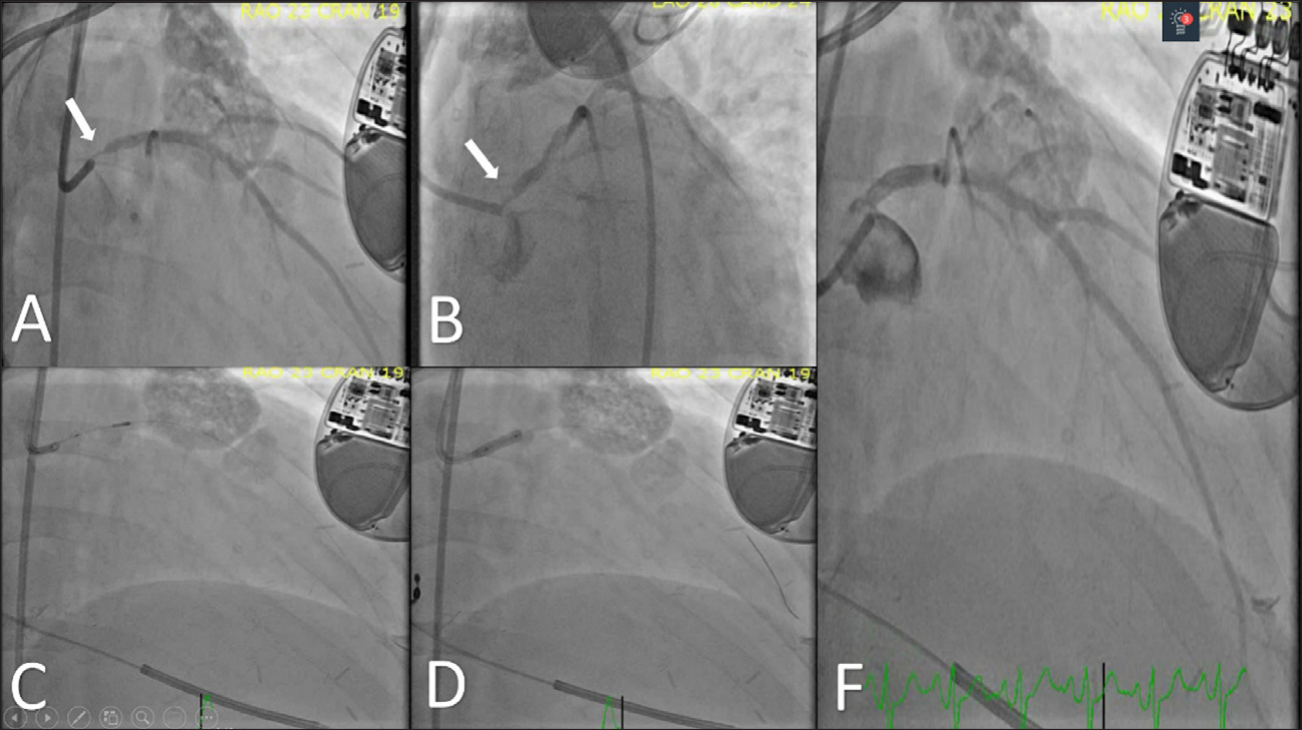
Percutaneous coronary intervention of a severe ostial left main coronary artery stenosis. (**A**, **B**) Severe (95%) stenosis of the ostial left main coronary artery; (**C**) intravascular ultrasound to assess lesion and plan stent sizing; (**D**) deployment of a 3.5 × 12 mm Xience (pre-dilated with a compliant balloon and postdilated with a noncompliant 4.0 × 12 mm balloon); (**F**) final angiography showing 0% residual stenosis with TIMI 3 flow distally without complications. TIMI: Thrombolysis in Myocardial Infarction Score

On follow-up, the patient reported markedly improved chest pain and exercise capacity.

## Points to Remember

- Radiation heart disease can present with coronary atherosclerotic vascular disease and occurs, on median, 19 years after completing radiation therapy for Hodgkin’s lymphoma. The risk of disease is linearly correlated with the radiation dose received.^1^
- Other manifestations of radiation heart disease include myocardial disease (ie, nonischemic myocardial scar, restrictive cardiomyopathy), pericardial constriction, conduction system disease (ie, sick sinus syndrome, complete heart block), and valvular heart disease with classically aortomitral curtain thickening and calcification.^2^
- Patients with a known diagnosis of nonischemic cardiomyopathy can have concomitant or new-onset coronary artery disease (CAD), which needs to be appropriately investigated.
- Risk factors for radiation heart disease can be broken down into therapy-related risk factors and patient-related risk factors. The classic presentation of radiation-induced CAD is a young patient with little or no risk factors.
- The pathophysiology of radiation-induced CAD includes endothelial effacement, chronic inflammation, oxidative stress, and fibrosis.^2^
- Prevention of radiation heart disease is crucial and includes minimizing radiation dose, field and proximity of radiation to the heart, and cardiotoxic chemotherapy. In addition, cardiac risk factor modification and an active lifestyle are imperative.^2^
- The patient-related risk factors include a history of classic CAD risk factors and younger age at the time of receiving chemotherapy.^2^
- The typical distribution for radiation-induced CAD includes ostial stenosis (as seen in our case) with a proclivity for the left main, right coronary artery, and proximal left anterior descending artery. The lesions typically are long, concentric, smooth, and tubular.^3^
- Patients with radiation-induced left main disease or left anterior descending stenosis may be treated with percutaneous coronary intervention or coronary artery bypass graft surgery (CABG). CABG can be complicated due to extensive radiation-induced scarring of surrounding tissue, including the pericardium, skin, and subcutaneous tissue; fibrosing, scarred, or stenosed arterial and venous conduits; concomitant radiation-induced interstitial lung disease; and porcelain aorta limiting aortic clamping for cardiopulmonary bypass.^4^
